# Humidity Sensors Based on ZnO-BiFeO_3_ Nanocomposites

**DOI:** 10.3390/s26134034

**Published:** 2026-06-25

**Authors:** Rachida Douani, M’Hand Oughanem, Hayat Hammouche, Malika Saidi, Nouara Lamrani, Yannick Guhel, Ahcène Chaouchi, Bertrand Boudart, Saliha Rabehi

**Affiliations:** 1 Laboratoire de Chimie Appliquée et Génie Chimique, Faculté des Sciences, UMMTO de Tizi Ouzou, Tizi-Ouzou 15000, Algeria; rachida.douani@ummto.dz (R.D.); mhand.oughanem@ummto.dz (M.O.); malika.saidi@ummto.dz (M.S.); nouara.lamrani@ummto.dz (N.L.); ahcene.chaouchi@ummto.dz (A.C.); 2 Laboratoire de Chimie Appliquée et Génie Chimique, Faculté de Génie Electrique, UMMTO de Tizi Ouzou, Tizi-Ouzou 15000, Algeria; hayat.hammouche@ummto.dz (H.H.); saliha.rabehi@ummto.dz (S.R.); 3 Université Caen Normandie, ENSICAEN, CNRS, Normandie University, GREYC UMR6072, F-14000 Caen, France; yannick.guhel@unicaen.fr

**Keywords:** sensor, humidity, sensitivity, ZnO-BiFeO_3_, nanocomposite

## Abstract

The aim of this study was to investigate the impact of ZnO nanoparticles on the humidity-sensing properties of BiFeO_3_ nanoparticles. BFO, ZnO, and x% ZnO-BiFeO_3_ nanoparticles were synthesized using chemical processes and then analyzed by Scanning Electron Microscopy (SEM), X-Ray Diffraction (XRD), and Raman spectroscopy. The electrical capacitance of the sensors was measured using an impedance meter over a Relative Humidity (RH) range of 17 to 94% at room temperature and at an applied frequency of 100 Hz. This paper demonstrates that adding zinc oxide (ZnO) to bismuth ferrite (BFO) materials significantly improves the humidity response of BFO-based sensors. Indeed, a response of 2.8 × 10^6^% was achieved for 20% ZnO-BFO-based sensors, compared with 5.8 × 10^3^% for a pure BFO-based sensor. At the same time, a low hysteresis effect and excellent long-term stability were observed. In conclusion, the addition of ZnO nanoparticles provides excellent humidity-sensing properties to the BFO material, thereby contributing to its wide range of applications.

## 1. Introduction

Humidity sensors play a crucial role in various aspects of human life. They facilitate the monitoring and regulation of humidity levels in a wide range of fields, such as cryogenics and food processing, medical instrumentation, antique conservation, electronics, and chemical materials [1,2].

Various sensing materials have been used in the development of humidity sensors, namely polymers [3,4], inorganic/organic composites [5,6], ceramic materials [7,8], cellulose-based materials [9], clay-based materials such as halloysite nanotubes [10], attapulgite [11], and sepiolite nanofibers [12]. Metal oxides, in their pure form or as composites, are more commonly used in the development of sensing layers for humidity sensors due to their low cost and ease of fabrication. These materials have physicochemical properties that are perfectly suited to this type of application, such as a large specific surface area, chemical and thermal stability, and numerous sites for the adsorption of water molecules [13,14]. Humidity sensors can operate according to different transduction mechanisms, such as resistive [15], capacitive [16], impedance [17], optical [18], frequency [19], and electrochemical sensors [20]. Capacitive humidity sensors are particularly attractive because of their low power consumption, simple structure, good stability, and suitability for room-temperature operation [2].

Bismuth ferrite BiFeO_3_ perovskite is a p-type semiconductor and an archetypal room temperature multiferroic material that has attracted significant research interest in recent years. It possesses various properties, including a narrow band gap (ranging from 2.2 to 2.8 eV at 300 K), excellent chemical stability [21], and the coexistence of simultaneous ferroelectric and magnetic parameters. BiFeO_3_ has been widely investigated in sensing applications, particularly in gas sensing applications such as Liquefied Petroleum Gas (LPG) [22], ammonia [23], SO_2_ [24], ethanol [25,26], and acetone [27,28,29]. However, to the best of our knowledge, there are relatively few literature reports on the humidity-sensing properties of BiFeO_3_ nanoparticles [30,31,32]. Indeed, the capacitance of the BFO film-based sensor increases from 25 to 1410 pF when the relative humidity varies from 30% to 90% [32]. Likewise, the humidity sensitivity of the BFO nanoparticle-based sensor rises from 0.7 up to 4652% for an RH ranging from 34% to 92% [31].

In a previous study [31], we highlighted that adding carbon fibers (CF) to BiFeO_3_ nanoparticles significantly improves the humidity-sensing properties. Thus, the BFO/CF nanocomposite-based sensor exhibits a humidity sensitivity of up to 12640% for RH varying up to 92%, while the maximum sensitivity of a BFO-based sensor does not exceed 4652% over the same relative humidity.

We believe that incorporating materials other than CF into BFO nanoparticles may also increase their sensitivity to humidity. In terms of material choice, zinc oxide is an environmentally friendly n-type semiconductor that demonstrates enhanced electron mobility and reduced recombination losses when compared to other wide bandgap oxides [33]. Moreover, it possesses a combination of properties necessary for an ideal sensor material, including a bandgap of 3.37 eV, a high exciton binding energy of 60 meV, a high concentration of oxygen vacancies, and excellent chemical and thermal stability. Additionally, ZnO materials offer the advantage of being inexpensive, easy to prepare, and suitable for mass production [34,35]. Various types of ZnO morphologies have been widely utilized as gas and humidity sensors, including nanorods [36,37], nanowires [38,39], nanoplatelets [40], nanotubes [41], and nanoparticles [42,43,44,45]. However, some humidity sensors based on pure ZnO may exhibit slow response times, poor linearity [46], poor response in low humidity environments, and a large hysteresis [47,48].

In the present study, ZnO nanoparticles were employed as an alternative additive to further improve the sensing properties of BiFeO_3_ perovskite. The humidity-sensing properties of the BFO and x% ZnO-BFO-based sensors (where x = 10 and 20) were evaluated at room temperature over a range of 17% to 94% relative humidity. The results showed that the inclusion of ZnO nanoparticles greatly enhanced the humidity-sensing performance of pure BFO particles.

## 2. Materials and Methods

### 2.1. Preparation of BFO, ZnO and x% ZnO-BFO Particles

Chemical reagents of high analytical purity were utilized in this study. The chemical reagents used included iron (III) nitrate nanohydrate (Fe(NO_3_)_3_, 9 H_2_O) (99%), bismuth nitrate pentahydrate (Bi(NO_3_)_3_, 5 H_2_O) (99%), nitric acid HNO_3_ (69%), citric acid monohydrate (C_6_H_8_O_7_, H_2_O) (99.5%), zinc acetate monohydrate Zn (CH_3_COO)_2_, H_2_O), sodium hydroxide (NaOH), and deionized water. All chemical reagents were purchased from Sigma-Aldrich (St. Louis, MO, USA).

BiFeO_3_ nanoparticles were prepared using a standard sol–gel method. Initially, a 0.105 M aqueous solution of bismuth nitrate was prepared by dissolving 2.5468 g of (Bi(NO_3_)_3_, 5H_2_O) in 50 mL of deionized water. Subsequently, 20 mL of nitric acid was gradually added until a clear solution was obtained. Then, 2.02 g of (Fe(NO_3_)_3_, 9H_2_O) was introduced to this solution. Finally, 5.0708 g of citric acid was incorporated into the mixture and stirred at 80 °C until a gel formed. This gel was then dried at 120 °C. The resulting xerogel was calcined at 350 °C for 2 h, followed by an additional 2 h at 700 °C.

**Figure 1:**
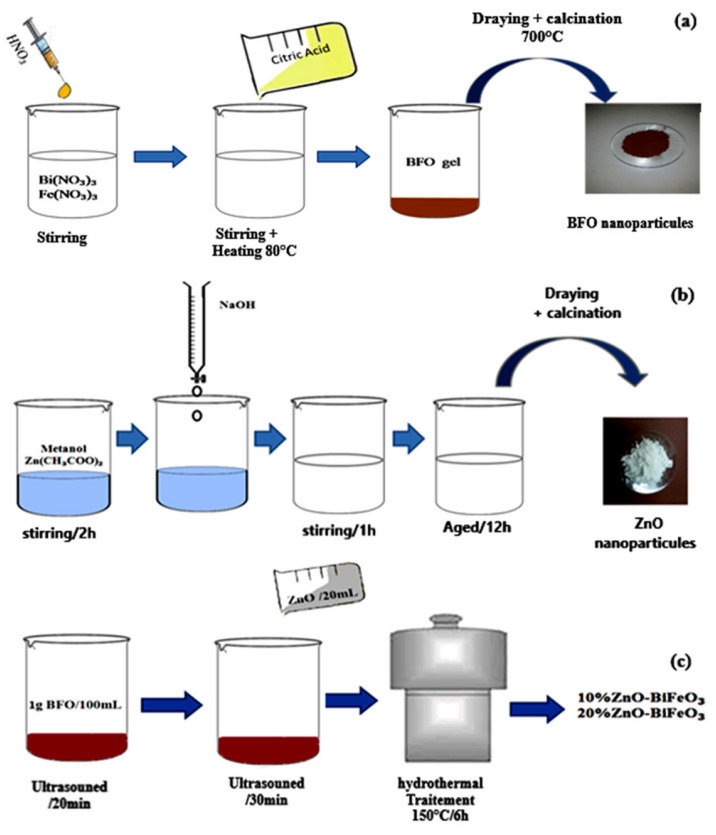
Different steps in the synthesis of: (**a**) BFO nanoparticles; (**b**) ZnO nanoparticles; (**c**) x% ZnO-BFO composites.

ZnO nanoparticles were prepared using the co-precipitation method. Initially, 1.1 g of zinc acetate (Zn(CH_3_COO)_2_, H_2_O) was dissolved in 100 mL of methanol (CH_3_OH). After stirring for 2 h, a solution of sodium hydroxide (1M) was added dropwise until a pH of 9 was reached. The mixture was stirred for 1 h and then allowed to stand for 12 h. The precipitate was washed several times with distilled water, and the resulting powder was dried at 100 °C for 12 h. Subsequently, the powder was calcined in two stages (at 250 °C/2 h, followed by 550 °C/2 h, with a heating rate of 5 °C/min).

x% ZnO-BFO nanocomposites were synthesized using the hydrothermal process with pre-prepared ZnO and BFO nanoparticles. An appropriate mass of ZnO was dispersed in 25 mL of distilled water, and the resulting suspension was then added to an aqueous suspension of BFO (100 mL, 10 mg/mL) under ultrasonic conditions. The resulting mixture was transferred to a 200 mL autoclave, heated at 150 °C for 6 h, and then naturally cooled to room temperature. Finally, the x% ZnO-BFO composites were washed with deionized water and dried at 100 °C for subsequent use. The various synthesis steps are illustrated in Figure 1.

### 2.2. Material Characterizations

The morphology and dispersity of the prepared particles (BFO, ZnO, and x% ZnO-BFO) were investigated using a JEOL SEM7200 microscope (JEOL, Paris, France). The crystallographic structure and the effect of ZnO addition on the crystallinity of BFO nanoparticles were studied using a Panalytical Empyrean X-ray diffractometer (Malvern Panalytical, Paris, France) (Cu Kα (1.5406 Å) at 45 kV). The synthesized particles were also characterized by Raman spectroscopy using a Renishaw InVia spectrometer (Renishaw, Bristol, UK) with a visible laser at a wavelength of 632.8 nm.

### 2.3. Preparation of the Sensors

The sensors under investigation consist of copper spiral electrodes with a surface area of 180 mm^2^, onto which films of sensitive materials are deposited. The preparation process of these sensors is outlined as follows: a homogeneous solution was prepared by dispersing 0.2 g of the synthesized powders (BFO or x% ZnO-BFO) in approximately 3 drops of polyvinyl alcohol (PVA), which was used to ensure the adhesion of the powders to the surface of the structure. The mixture was then applied to the pre-cleaned electrodes and dried at 160 °C for 1 h to remove excess PVA in the sensor matrix.

### 2.4. Sensing Measurements

**Figure 2:**
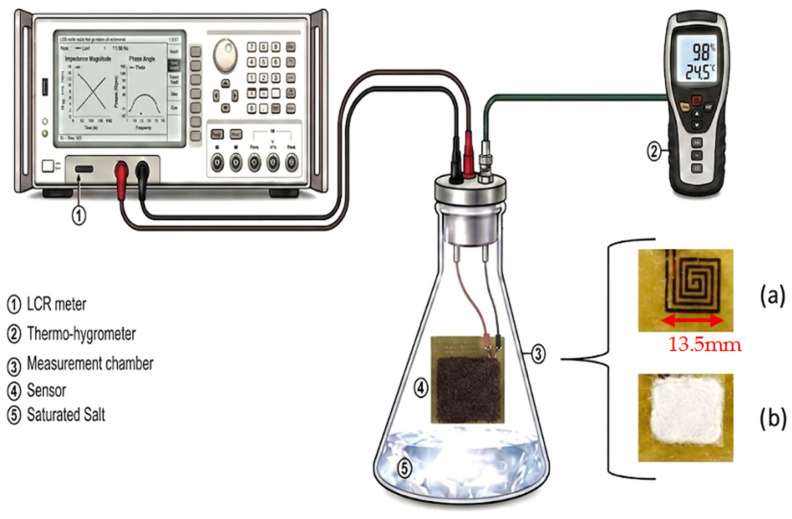
Schematic representation of the electrical measurement system. Example of the sensor (**a**) before and (**b**) after the sensitive layer deposition.

The sensors were tested at room temperature (approximately 25 °C) and at atmospheric pressure in a humidity-controlled environment, as depicted in Figure 2.

The electrical measurements were performed using an LCR HP 2484A impedance-meter, as shown in Figure 2, over a frequency range of 100 Hz to 1 MHz, with an applied voltage of 1 Volt. The humidity sensitivity of the sensors was characterized at different relative humidity levels, obtained by introducing various salts into the measurement systems.

## 3. Results and Discussion

### 3.1. Morphological and Structural Characterizations

**Figure 3:**
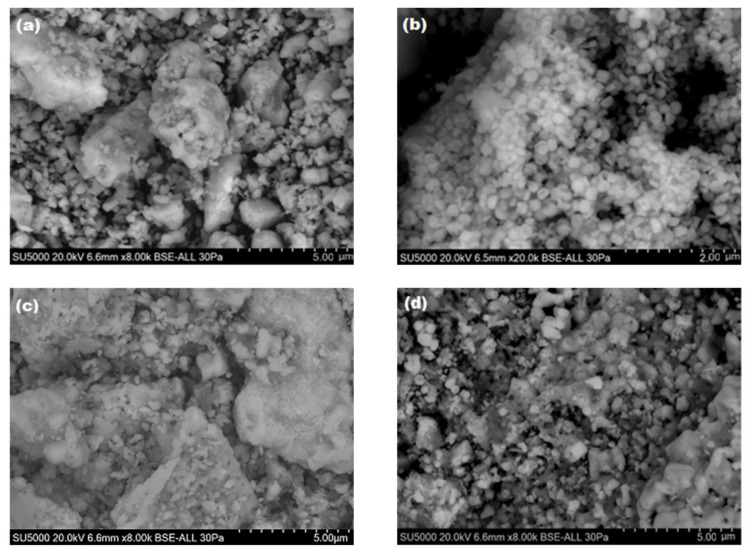
SEM micrographs of (**a**) pure BFO particles, (**b**) pure ZnO particles, (**c**) 10% ZnO-BFO composite, and (**d**) 20% ZnO-BFO composite.

Figure 3 shows scanning electron microscope images of BFO, ZnO, and x% ZnO-BFO powders. As shown in Figure 3a, the BFO particles appear as aggregates, probably due to the high surface reactivity of the material, which promotes particle agglomeration. By comparison, the SEM micrograph of the ZnO powder shown in Figure 3b reveals spherical nanoparticles with a relatively uniform distribution. Finally, SEM observations of the x% ZnO-BFO composite powder shown in Figure 3c,d reveal a homogeneous distribution of the particles, which are more widely dispersed than those in pure BFO.

**Figure 4:**
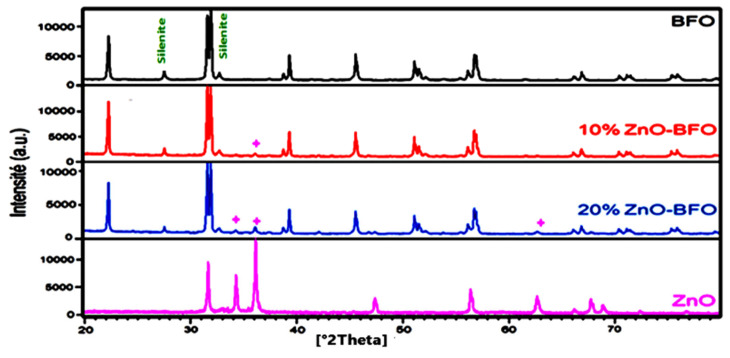
X-ray diffraction patterns of BFO, ZnO, and x% ZnO-BFO powders.

To investigate the effect of the ZnO incorporation on the crystallinity of BFO during the synthesis of x% ZnO-BFO composite, X-ray diffraction patterns of BFO nanoparticles and x% ZnO-BFO composite powders are shown in Figure 4.

The X-ray diffraction peaks observed in the ZnO powder pattern are in good agreement with the standard space group (P63mc) wurtzite diffraction pattern (JCPDS No [01-089-7102]). However, no secondary phase was detected in the patterns. As for the BFO nanoparticles, they crystallize in a hexagonal structure with space group R3c (JCPDS No. 92-210-2910). The peaks appearing at 2θ = 27.71° and 32.904° correspond to sillenite (Bi_25_FeO_38.96_) (JCPDS No. 96-901-1269), which is identified as the secondary phase. Finally, the diffractograms of the x% ZnO-BFO composite powders show the same diffraction peaks as those observed in the diffraction profile of the BFO powder. It can therefore be concluded that the zinc oxide addition to BFO perovskite and the hydrothermal treatment do not alter the crystallographic structure of BFO. The presence of ZnO in the composite is confirmed by the peaks appearing at 2θ = 34.33° and 36.17°. As expected, these two peaks are more intense for x = 20% than for x = 10%.

**Figure 5:**
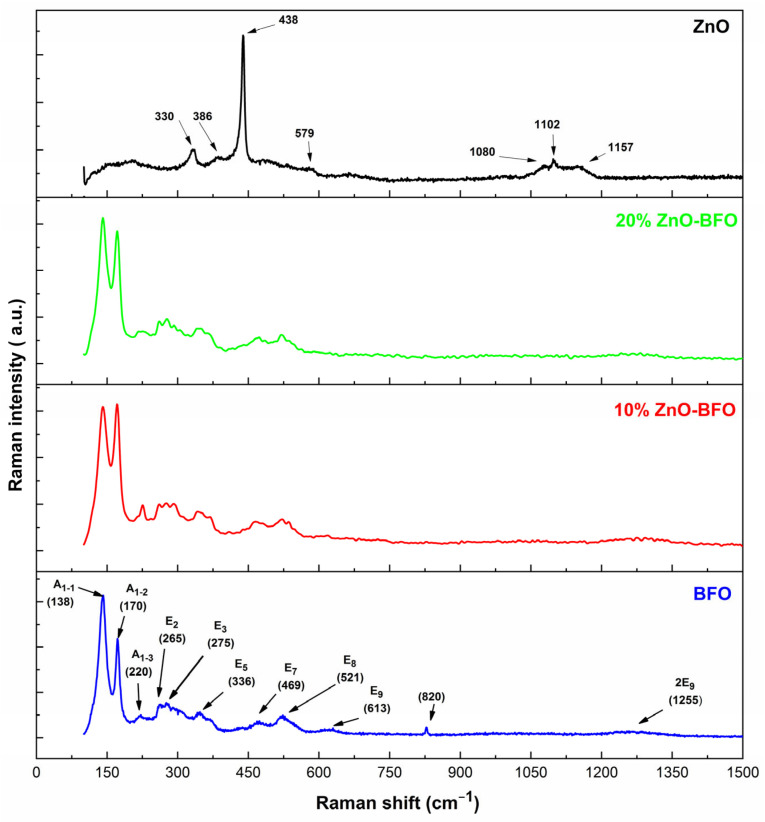
Raman spectra of BFO, ZnO, and BFO-x% ZnO powders at room temperature.

In order to confirm these results, these materials were characterized by Raman spectroscopy. Figure 5 shows the Raman spectra of BFO, 10% ZnO-BFO, 20% ZnO-BFO, and ZnO. For BFO, the determination of the vibrational modes is based on a comparison of the experimental results obtained with those of previous studies on the R3c structure of BFO [49,50,51,52,53,54]. Ten active Raman modes were observed. The peaks observed at 138, 170, 220 cm^−1^ are attributed to the A_1-1_, A_1-2,_ and A_1-3_ vibrational modes, respectively, of the Bi-O band. The peaks observed at 265, 275, 336, 469, 521, 613, and 1255 cm^−1^ are associated, respectively, with the E_2_, E_3_, E_5_, E_7,_ E_8_, E_9,_ and 2E_9_ vibrational modes of the Fe-O band. In addition, the peak observed at 820 cm^−1^ is probably due to the presence of the secondary phase (sillenite) previously identified by X-ray diffraction.

The Raman spectrum of ZnO exhibits the vibrational modes characteristic of the hexagonal wurtzite structure [55,56,57]. The peak at around 330 cm^−1^ is attributed to the E_2_ (high)-E_2_ (low) mode, which corresponds to second-order Raman scattering. The peak at 386 cm^−1^ is attributed to the A_1_ (TO) mode, and that at 438 cm^−1^ is attributed to the E_2_ (high) mode, both of which are characteristic of ZnO in the wurtzite structure [58]. The peak observed at 579 cm^−1^ is attributed to the A_1_ (LO) vibration mode, indicating the presence of electrostatic order in the system. This mode is associated with the formation of structural defects such as oxygen vacancies (VO°°), vacant zinc sites (VZn″), or impurities [57]. The peaks appearing at 1080 cm^−1^ and 1102 cm^−1^ are attributed to the combination of A_1_ and E_2_ modes. Finally, the peak at 1157 cm^−1^ is attributable to vibrational mode 2 A_1_ (LO).

For ZnO-BFO composites, the Raman spectra are dominated by the characteristic modes of BFO, and the same Raman bands are observed in all these cases. These results are surprising, as no Raman modes are observed in ZnO.

**Figure 6:**
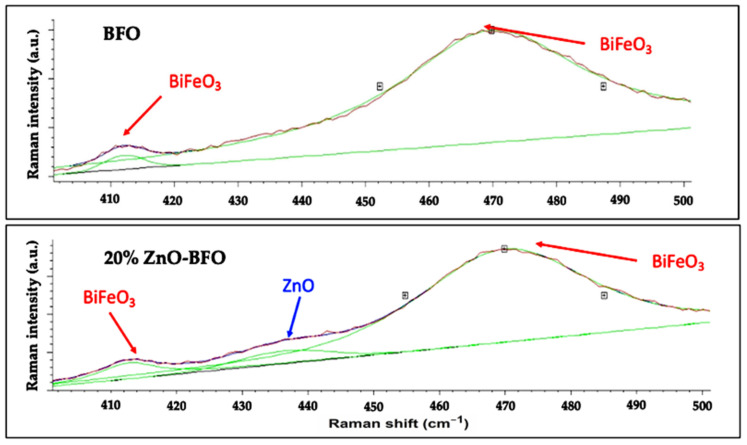
Comparison between the experimental and fitted Raman spectra of BFO and 20% ZnO-BFO powders at room temperature. The green curves represent the baseline and the three peaks used to perform the fit.

That is why we have precisely characterized these powders over a range between 400 and 500 cm^−1^. The Raman peak attributed to ZnO, which has the highest intensity, is located at around 438 cm^−1^, as shown in Figure 6.

We fitted the experimental Raman spectra of 0% ZnO-BFO and 20% ZnO-BFO powders using a Lorentzian and Gaussian model, taking into account two Raman bands at 410 and 469 cm^−1^ attributed to the BFO powder [59,60,61], as well as a Raman band at 438.7 cm^−1^ associated with the ZnO powder [55,56,57]. There is a good match between the experimental spectra (red curves) and the fitted spectra (blue curves); it is difficult to distinguish between these two curves as they overlap perfectly.

Although the intensity of the Raman peak attributed to ZnO is low, it nevertheless indicates that ZnO is present in the 20% ZnO-BFO powder, unlike the 0% ZnO-BFO powder. Furthermore, this finding is consistent with the XRD characterizations presented earlier in this article.

### 3.2. Electrical Characterizations

**Figure 7:**
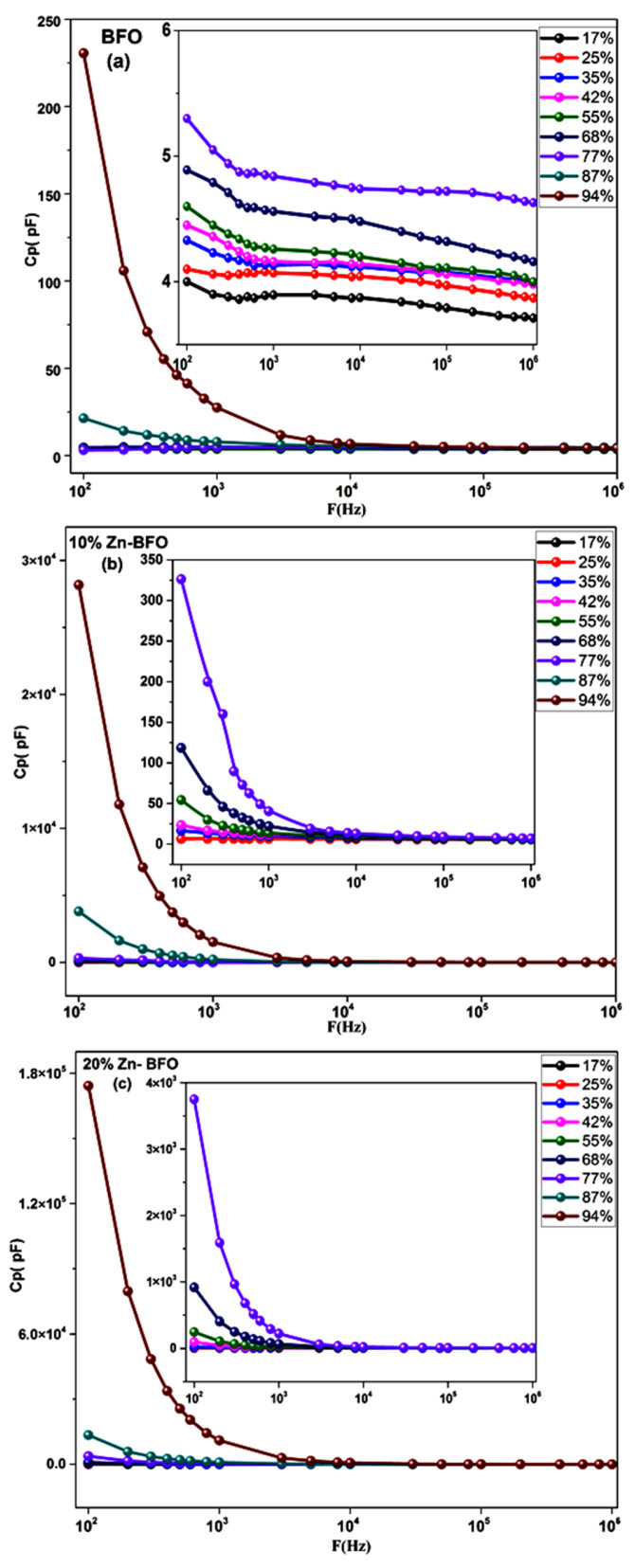
Evolution of capacitance versus frequency at RH values varying between 17% and 94% for BFO (**a**), 10% ZnO-BFO (**b**), and 20% ZnO-BFO (**c**) based sensors.

The variation in capacitance (Cp) of BFO and x% ZnO-BFO-based sensors as a function of frequency (F) has been plotted for a humidity range of 17–94%, as shown in Figure 7. A decrease in Cp is thus observed when F changes from 10^2^ to 10^6^ Hz, regardless of the relative humidity, for the BFO-based sensor (Figure 7a). However, the effect of frequency appears to be more pronounced when the RH is high. For example, a decrease in Cp from 4 and 230 pF to 2 and 4 pF is observed in this frequency range for RH = 17% and 94%, respectively. Furthermore, it appears that the variation in Cp is more pronounced when F varies between 10^2^ and 10^3^ Hz than between 10^3^ and 10^6^ Hz.

These results indicate that the dependence of Cp on relative humidity is very weak above 10 kHz, as is often reported in the literature for the metal oxide sensors [62]. In this case, the electric field alternates too rapidly for the dipoles associated with the water molecules adsorbed on the surface of the sensor to reorient themselves. Consequently, these molecules cannot be effectively polarized during high-frequency electric field variations, leading to a reduction in the dielectric constant and a negligible response. This means that the capacitance is practically independent of humidity and depends only on the electrical properties of the sensitive layer [63].

The same trends are observed for the 10% ZnO-BFO and 20% ZnO-BFO-based sensors (Figure 7b,c). Nevertheless, the Cp values are significantly higher for the sensor based on a 20% ZnO-BFO composite than for those based on a 10% ZnO-BFO composite and for the BFO material, regardless of F and RH values. Similarly, it is interesting to note that the 20% ZnO-BFO-based sensor seems to be more sensitive to humidity than the other two sensors, particularly at low frequencies such as 100 Hz, for example.

**Figure 8:**
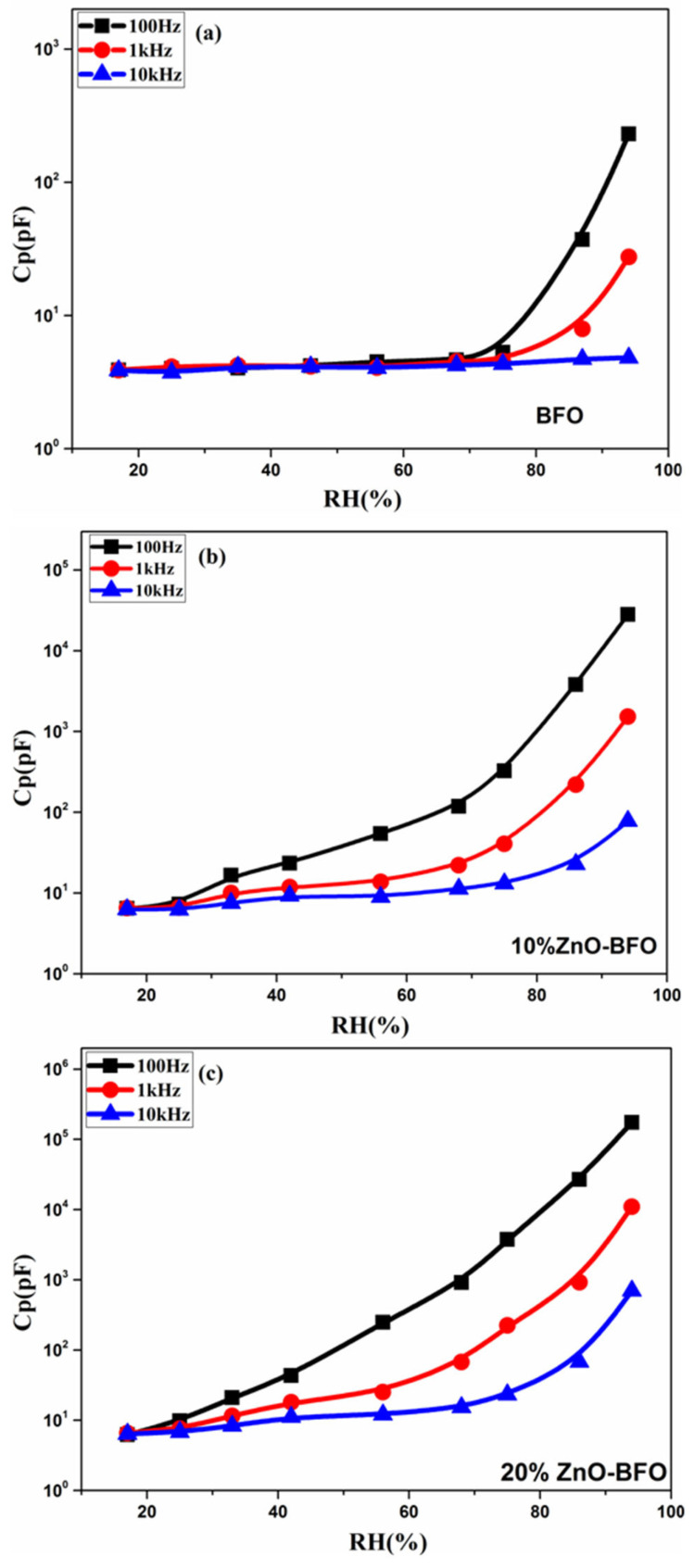
Evolution of capacitance versus RH values for BFO (**a**), 10% ZnO-BFO (**b**), and 20% ZnO-BFO (**c**) based sensors when the measurements are made at a frequency of 100 Hz (squares), 1 kHz (circles), and 10 kHz (triangles).

To confirm these results, Figure 8 presents the evolution of Cp versus RH for BFO, 10% ZnO-BFO, and 20% ZnO-BFO-based sensors.

Thus, the Cp values are higher when measurements are carried out at a frequency of 100 Hz than when they are taken at 1 kHz, and the values obtained at 1 kHz are higher than those measured at 10 kHz, regardless of the RH range. Moreover, Cp varies between 68% and 94%, between 35% and 94% and between 25% and 94%, for BFO, 10% ZnO-BFO and 20% ZnO-BFO-based sensors, respectively. In addition, the variation in Cp with relative humidity is more pronounced at a frequency of 100 Hz for all sensors, as already seen in Figure 7.

It can be concluded that incorporating ZnO particles into the BFO material improves the sensitivity to humidity across all RH levels and increases sensitivity at low RH levels. For example, the 20% ZnO-BFO-based sensors detect moisture at RH = 25%, unlike those made solely with BFO (RH = 68%).

**Figure 9:**
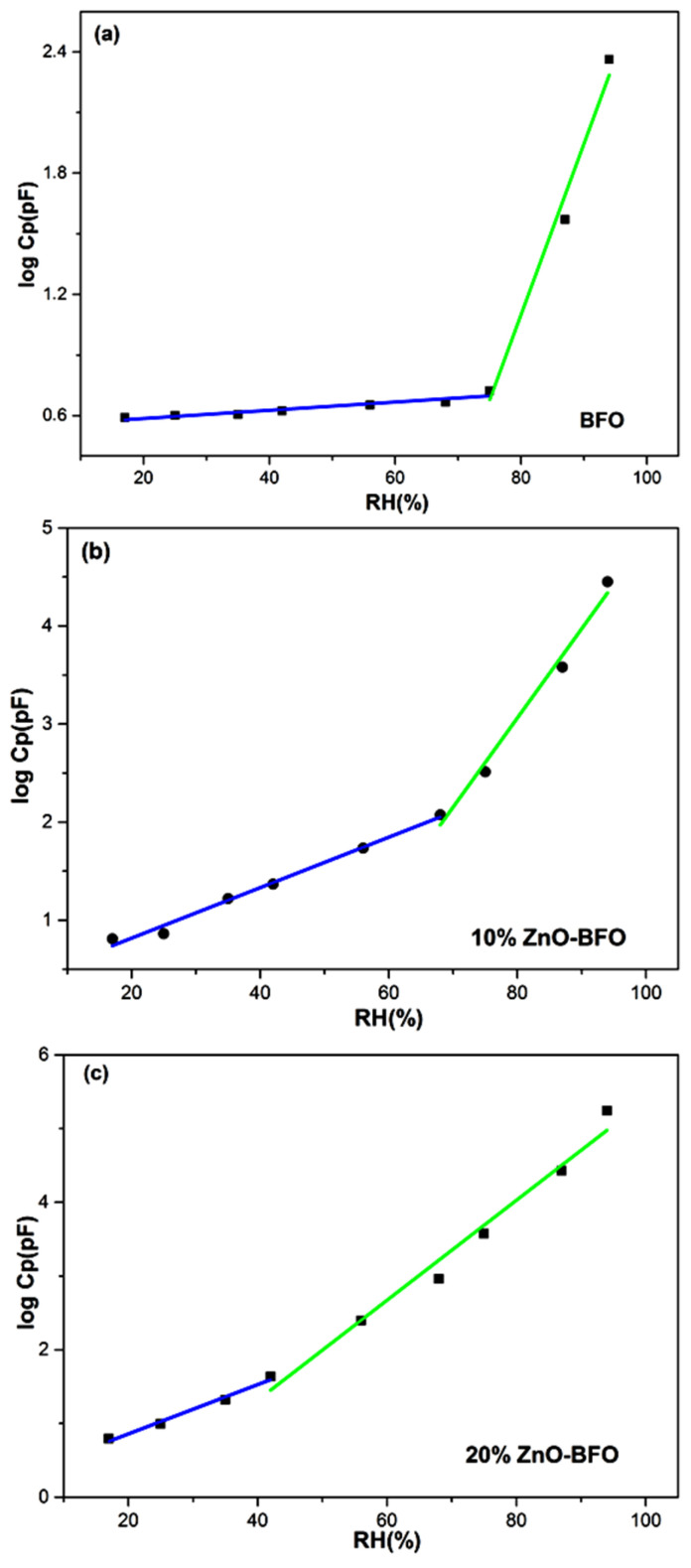
Linear fitted curves Log (Cp) versus RH (%) for BFO (**a**), 10% ZnO-BFO (**b**), and 20% ZnO-BFO (**c**) based sensors when the measurements are taken at a frequency of 100 Hz.

In any case, it can be seen that the increase in the Cp value is slow as RH rises from 17% to 68%, but then accelerates considerably between 68% and 94%. To confirm this phenomenon, Figure 9 shows the linear fit of Log (Cp) against relative humidity for the various sensors.

The results reveal two distinct linear relationships between Log (Cp) and relative humidity. An increase in the slope of the lines is observed for all sensors at humidity levels above their critical points: 75%, 68%, and 42% for BFO, 10% ZnO-BFO, and 20% ZnO-BFO-based sensors, respectively. This change in slope is likely due to the alteration in the mode of water molecule adsorption, transitioning from a chemisorbed monolayer at low RH levels to multilayer physisorption at high RH levels [64,65].

**Figure 10:**
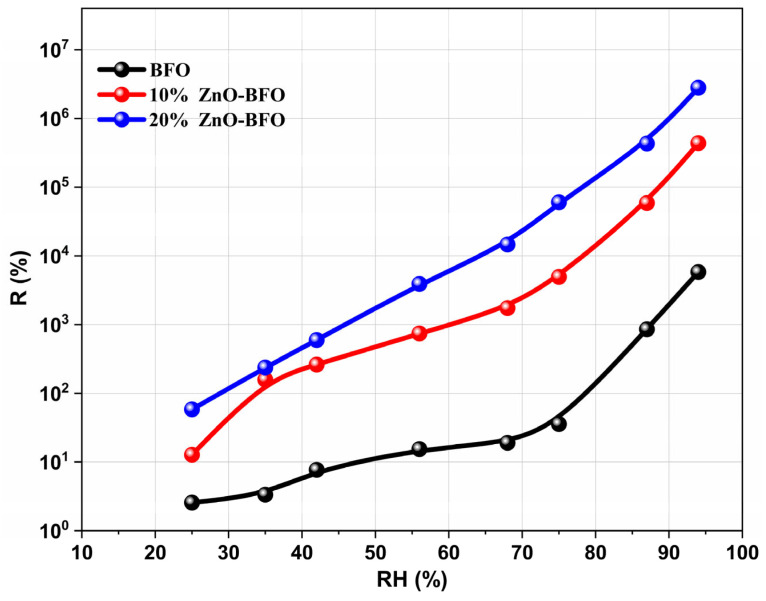
Response as a function of relative humidity for BFO, 10% ZnO-BFO, and 20% ZnO-BFO sensors. All measurements were taken at a frequency of 100 Hz.

Figure 10 shows the humidity response R(%) of the various sensors, which is calculated using the following equation:

(1) $$R\left(\%\right)=\frac{C_{x}{-C}_{17}}{C_{17}}\times 100$$

where $C_{17}$ and $C_{x}$ are the capacities measured at RH = 17% and x%, respectively.

We observe that R(%) of the 20% ZnO-BFO-based sensors is higher than that of the 10% ZnO-BFO-based sensors, which is itself superior to that of the BFO-based sensors, regardless of the RH. Indeed, R(%) increases from 2.6 to 5.8 × 10^3^%, from 12.7 to 4.3 × 10^5^%, and from 58.3 to 2.8 × 10^6^% as RH varies between 25% and 94% for BFO, 10% ZnO-BFO, and 20% ZnO-BFO sensors, respectively. These results demonstrate that the highest sensitivity is achieved for 20% ZnO-BFO sensors, with R(%) being 22.5 and 483 times higher at RH values of 25% and 94%, respectively, compared to the BFO sensors.

The improvement in response and sensitivity of the sensors, resulting from the increase in the amount of ZnO incorporated, can be attributed to the presence of oxygen vacancies generated by the ZnO, as shown in Figure 5. Indeed, the increase in the number of active sites on the material’s surface, caused by these oxygen vacancies, improves water adsorption and facilitates proton hopping at the surface, resulting in greater response to humidity.

The hysteresis effect is one of the key parameters used to assess the reliability of a humidity sensor; it is defined as the maximum difference between the sensor’s responses during an adsorption–desorption cycle.

Thus, the humidity hysteresis, denoted E (%), was calculated from the characteristic curves Cp = f(RH) measured for all sensors over a relative humidity range of 17% to 94%, using Equation (2) [48]:

(2) $$E\left(\%\right)=\frac{∆m}{{2Y}_{FS}}\times 100$$

where ∆m=Cp(adsorption)−Cp(desorption) and $Y_{FS}={Cp}_{\left(RH=94\%\right)}-{Cp}_{\left(RH=17\%\right)}$.

**Figure 11:**
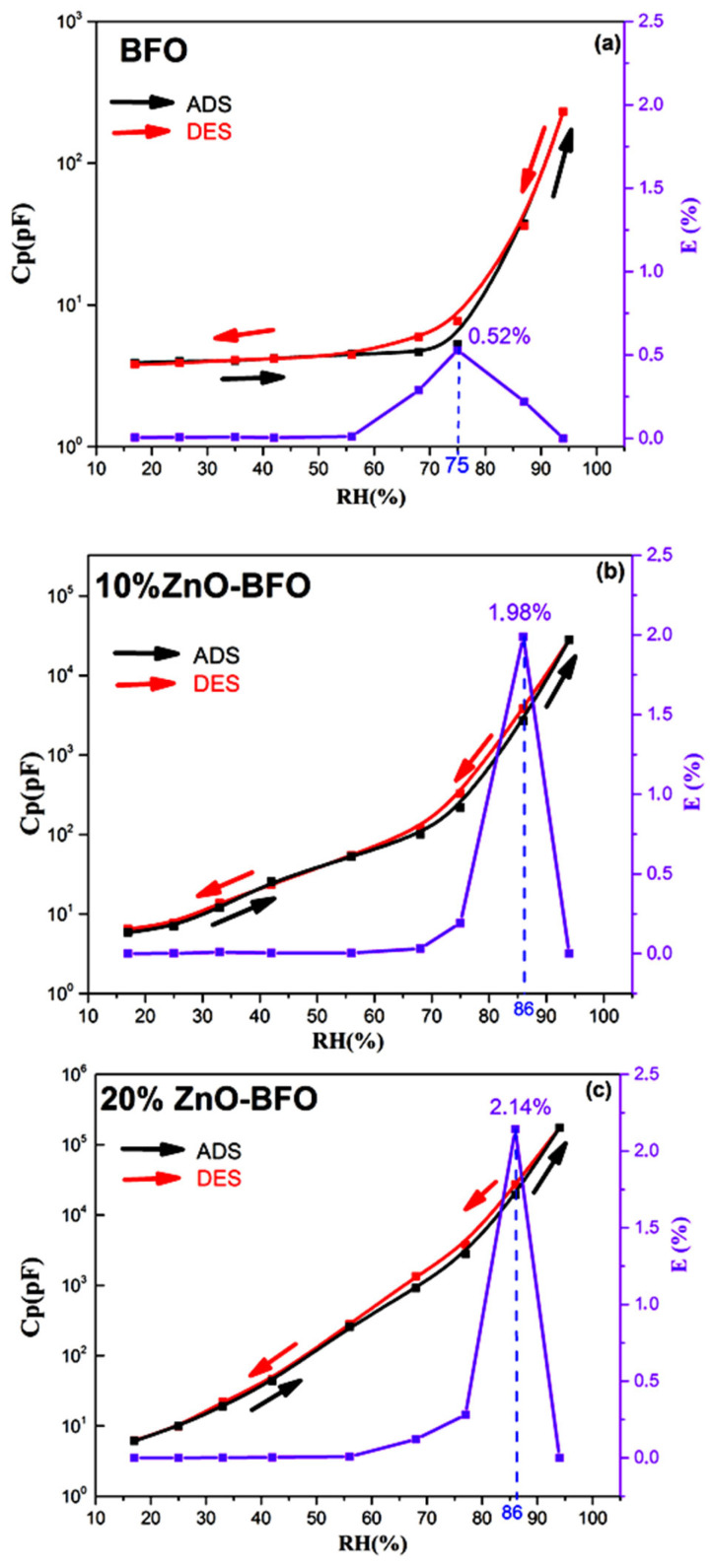
Variation in capacitance and hysteresis as a function of RH (%) during the adsorption–desorption processes of BFO (**a**), 10% ZnO-BFO (**b**), and 20% ZnO-BFO (**c**) sensors.

As shown in Figure 11, the sensors exhibit relatively narrow hysteresis loops, regardless of the sensors (<2.2%). This demonstrates that the addition of ZnO particles to BFO-based materials does not result in a significant hysteresis effect on the electrical behavior of the sensors, which is an advantage for humidity detection.

**Figure 12:**
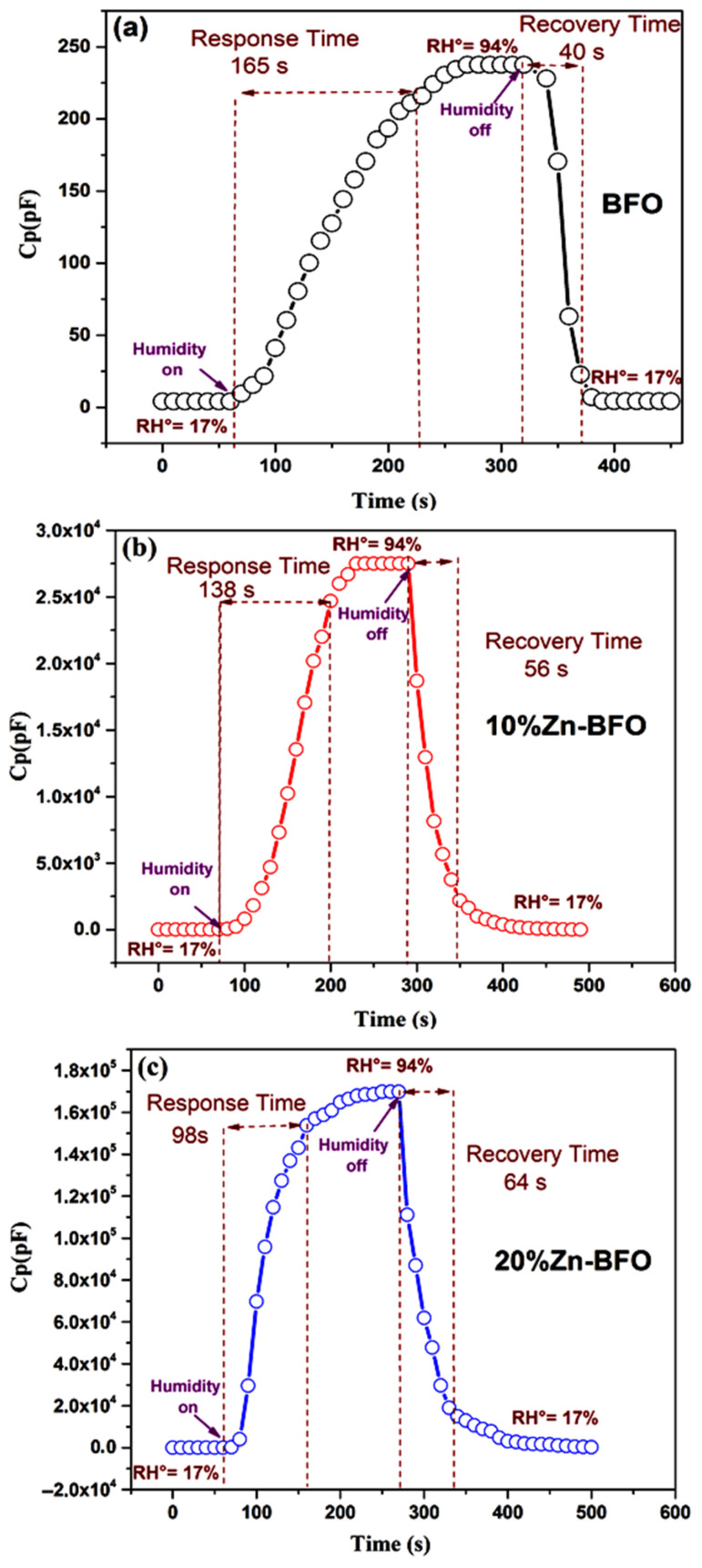
Response–recovery time graphs of BFO (**a**), 10% ZnO-BFO (**b**), and 20% ZnO-BFO (**c**) sensors.

Response and recovery times are important parameters for the implementation of humidity sensors. Figure 12, therefore, shows the capacitance response of the BFO, 10% ZnO-BFO, and 20% ZnO-BFO sensors over a relative humidity range of 17% to 94%. These measurements show that the response time of the sensors decreases from 165 to 98 s as the ZnO powder content in the x% ZnO-BFO composites increases from 0 to 20%. At the same time, the recovery time increases from 40 to 64 s.

These results are compared to those reported in the literature, as shown in Table 1.

It is difficult to compare the performance of the sensors shown in Table 1, as their humidity sensitivity, hysteresis effect, response times, and recovery times have not been measured under identical conditions. For example, the operating frequency, sensitive parameters, and humidity range are not always the same. It should nevertheless be noted that the 20% ZnO-BFO sensors are among those in Table 1 that exhibit the highest humidity sensitivities and the lowest hysteresis effects.

**Figure 13:**
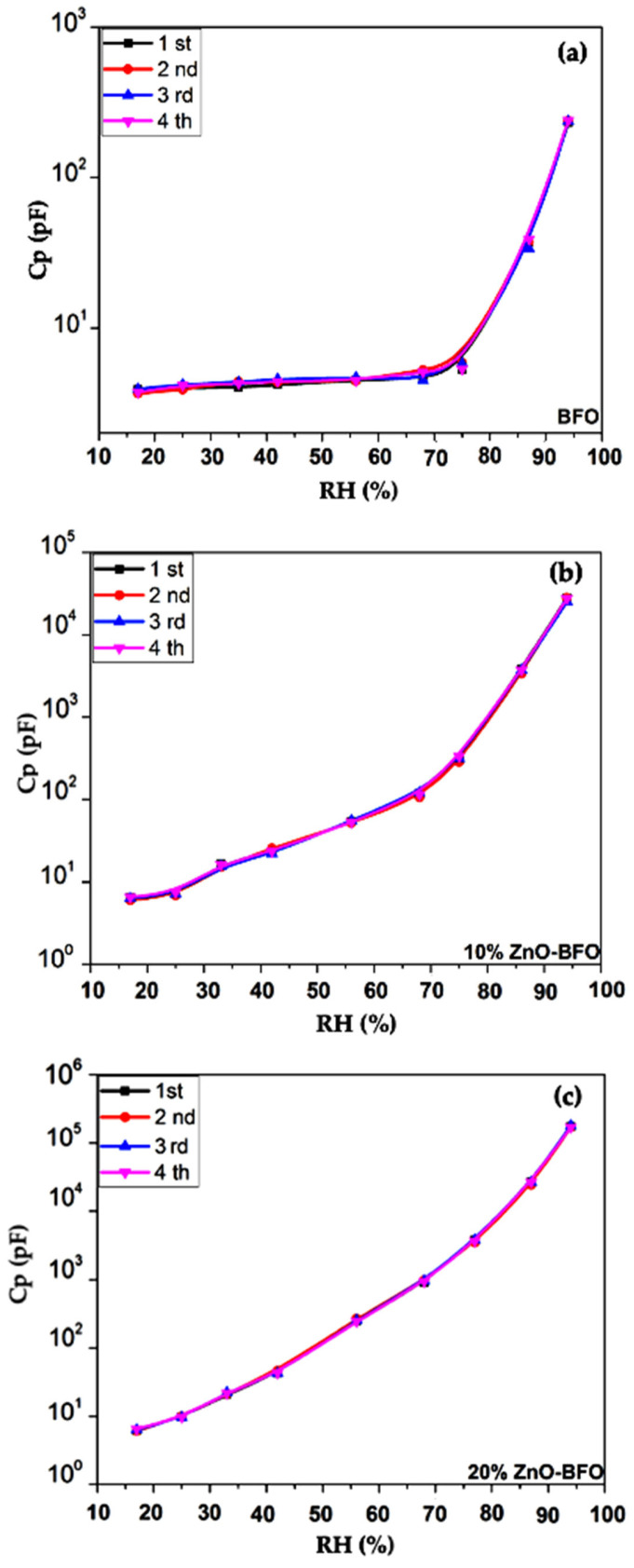
Repeatability of the capacitance response as a function of RH for BFO (**a**), 10% ZnO-BFO (**b**), and 20% ZnO-BFO (**c**) sensors when the measurements are carried out at a frequency of 100 Hz.

The performances of sensors must be repeatable regardless of their field of application. For these reasons, the measurements of the sensor capacitance across the entire relative humidity range were repeated four times, as shown in Figure 13.

Thus, we observe that the first, second, third, and fourth Cp (RH) curves are the same for BFO sensors (Figure 13a). The same tendencies are observed for 10% ZnO-BFO (Figure 13b) and 20% ZnO-BFO (Figure 13c) sensors. To confirm these results, the Relative Standard Deviation (RSD) of the x% ZnO-BFO sensors has been calculated by using Equation (3):

(3) $$RSD=\frac{Standard\;deviation\;of\;capacitance}{average\;capacitance}\times 100$$

Under these conditions, it can be stated that RSD is always less than 6.5%, regardless of the sensors used, which confirms that the capacitance response as a function of RH is repeatable.

As part of the long-term stability studies, measurements of capacitance as a function of relative humidity were taken every 10 days over a period of 200 days. The RSD results for various RH values ranging from 17% to 94% over time are presented in Table 2 and Table 3, for BFO sensors and 20% ZnO-BFO sensors, respectively.

The calculated fluctuations are very small. That is why the change in Cp over time for different sensors is plotted on a logarithmic scale.

**Figure 14:**
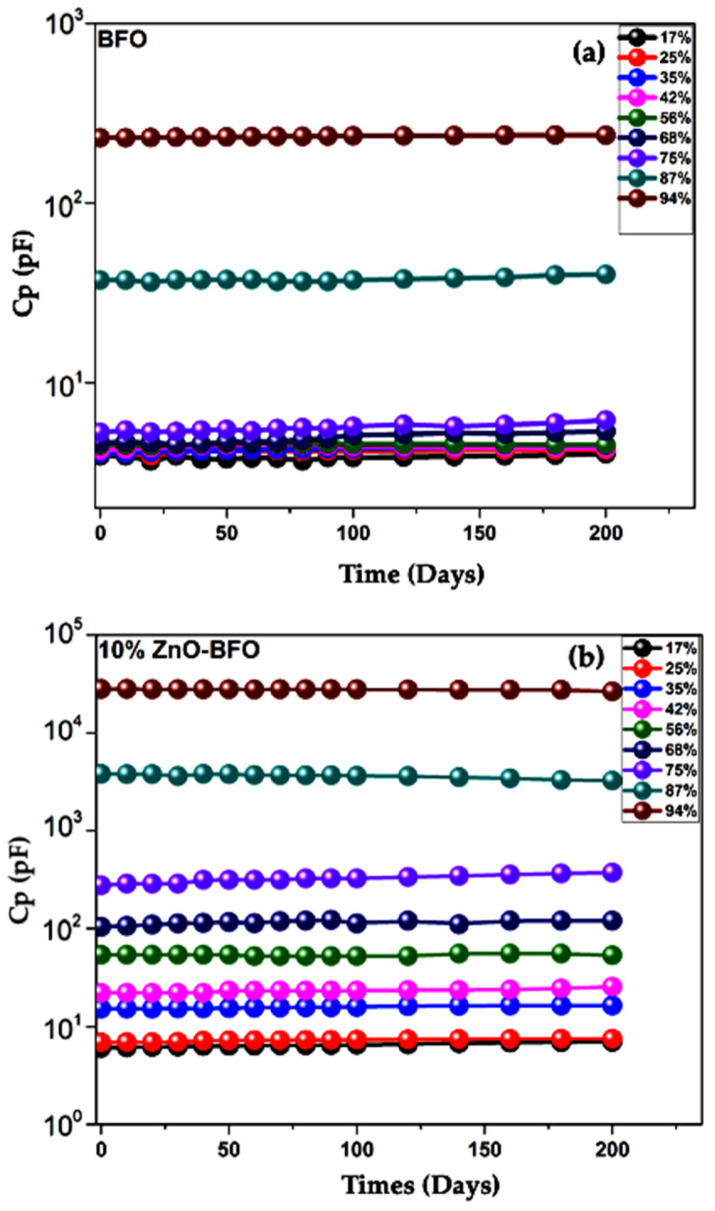

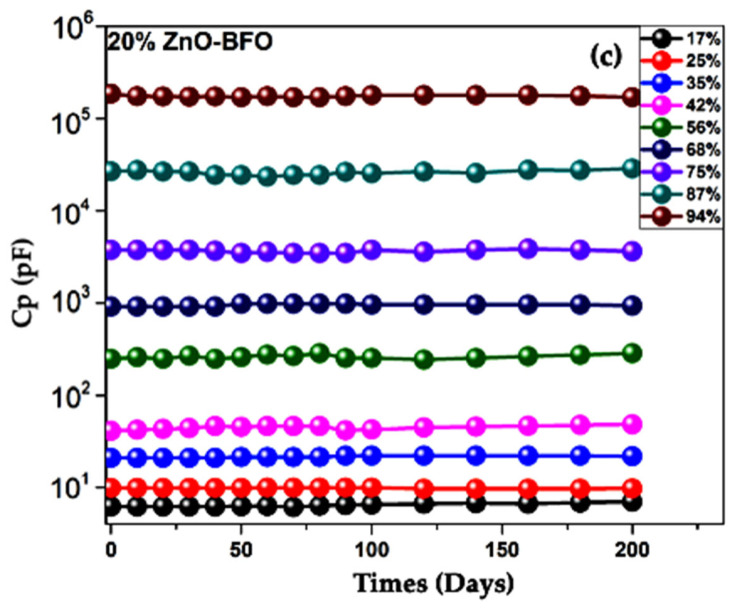
Long-term stability of BFO (**a**), 10% ZnO-BFO (**b**), and 20% ZnO-BFO (**c**) sensors for RH varying between 17% and 94%.

The results presented in Figure 14 demonstrate the remarkable stability of all the sensors, as evidenced by the minimal variation in their capacitance with respect to RH throughout the observation period.

The detection mechanism can be explained by the modification of the electrical response of the sensitive material due to the adsorption and desorption of water molecules. Complex electrical impedance (CEI), as demonstrated by Nyquist diagrams, allows for a thorough and comprehensive analysis of the operating mechanism of humidity sensors. It offers insights into the various physicochemical phenomena taking place at the sensor’s surface during water molecule adsorption.

**Figure 15:**
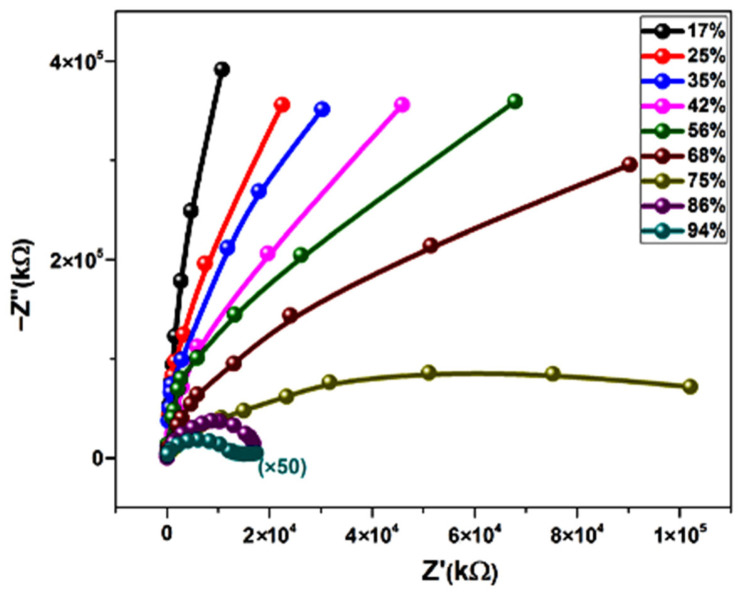
Complex electrical impedance plots of a pure BFO-based sensor for RH varying between 17% and 94%.

**Figure 16:**
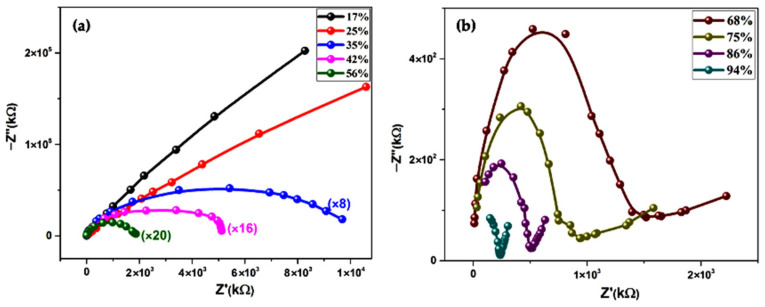
CEI plots of a 10% ZnO-BFO-based sensor at different humidity ranges: (**a**) 17–56% and (**b**) 68–94%.

**Figure 17:**
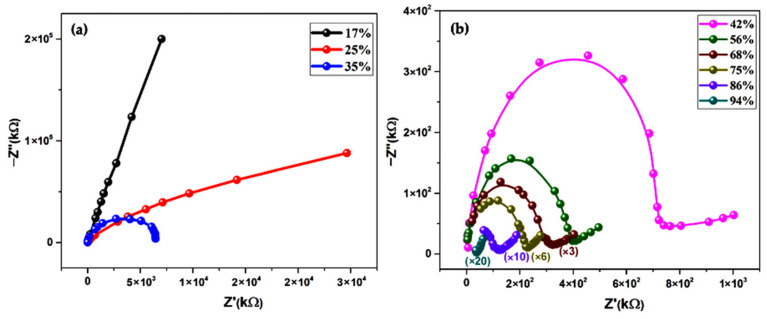
CEI plots of a 20% ZnO-BFO-based sensor at different humidity ranges: (**a**) 17–35% and (**b**) 42–94%.

The CEI of the sensors was measured at various relative humidity levels within the frequency range of 100 Hz to 1 MHz. The results are shown in Figure 15, Figure 16 and Figure 17, where (Z′) and (Z″) represent the real and imaginary parts of the impedance in the Nyquist diagram, respectively.

At RH = 17%, only an arc with a very large radius of curvature is observed (almost a straight line). This corresponds to the formation of a discontinuous layer of chemisorbed water molecules on the surface of the sensor [68]. When the sensor is exposed to low humidity, water molecules adsorb onto the active sites (Bi^3+^ and Fe^+3^) through a dissociative mechanism to form hydroxyl ions (OH^−^) and protons (H^+^). At this stage, ion conduction is impossible due to the discontinuity in the layer of adsorbed water molecules, so the sensor impedance is attributed to the intrinsic impedance of the sensing material [31].

As relative humidity increases, the radius of curvature of the arc decreases until a semi-circle is formed at 75% humidity. Within this range of relative humidity, a greater number of water molecules adsorb onto the surface, thereby forming a continuous film. This occurrence suggests the involvement of ionic conduction (proton hopping) in the charge transfer process, leading to a decrease in sensor impedance.

At RH = 94%, the semicircle is extended by a straight line. This phenomenon represents Warburg behavior, which is attributed to ion diffusion at the sensitive layer/electrode interface [69,70]. At high humidity levels, physisorbed multilayers form through the continuous adsorption of water molecules onto the material’s surface. The physical bonds between the water molecules in the outer layer gradually undergo a dissociative process, releasing protons (H^+^), as described by the Anderson and Parks model [71]:

H_2_O ⟶ H^+^ + OH^−^

The adsorbed water molecules then react with $H^{+}$ to form $H_{3}O^{+}$, as shown in the following reaction [72]:

H^+^ + H_2_O ⟶ H_3_O^+^

In fact, the outer water layer gradually exhibits liquid behavior, allowing free protons to move between adjacent water molecules through charge transport, following the Grotthuss mechanism:

H_2_O + H_3_O^+^ ⟷ H_2_O + H_3_O^+^.

Consequently, an increasing number of conductive proton transfer channels are formed, leading to enhanced polarization of the electric dipole moment in the sensitive material [63,73,74]. This polarization increase results in higher capacitance and, consequently, reduced impedance.

Compared with BFO-based sensors, those using x% ZnO-BFO composites show a significant reduction in electrical impedance, and a change in behavior is observed as the ZnO content of the composite increases. As shown in Figure 16 and Figure 17, the semicircle and Warburg line appear at lower humidity levels than with pure BFO.

However, at 94% RH, the Nyquist curves show only the line at the rear end of the semicircle, whilst the front end becomes invisible. This phenomenon can be explained by the greater number of physisorbed water molecules, which improve the conductivity of the sensing layer through the penetration of protons into the films (x% ZnO-BFO), leading to a significant decrease in the sensor’s impedance [68,75].

## 4. Conclusions

In this study, nanoparticles of zinc oxide, bismuth ferrite, and x% ZnO-BFO composites were synthesized. The results of XRD characterization showed that the BFO and ZnO nanoparticles crystallized in the hexagonal R3c and wurtzite phases, respectively. Furthermore, XRD measurements also demonstrate that the zinc oxide addition to the BFO perovskite and hydrothermal treatment do not alter the crystallographic structure of BFO. All these findings were confirmed by Raman spectroscopy.

Humidity sensors were fabricated using BFO-based materials, 10% ZnO-BFO and 20% ZnO-BFO, as the humidity-sensitive layers, and were then tested at relative humidity levels ranging from 17 to 94% at room temperature. It was found that the incorporation of ZnO into the BFO perovskite was beneficial for humidity detection. The maximum humidity response of 2.8 × 10^6^% was obtained for the 20% ZnO-BFO-based sensor. At the same time, a low hysteresis effect, reduced response time, good repeatability of the capacitive response as a function of RH, and excellent long-term stability were observed. The increase in humidity sensitivity is explained by the presence of oxygen vacancies generated by the ZnO.

For these reasons, we can conclude that the incorporation of ZnO into BFO-based materials represents a promising alternative for improving the humidity-sensing properties of BFO-based sensors.

## Figures and Tables

**Table 1:** Comparison of the humidity detection characteristics of BFO and x% ZnO-BFO-based sensors with those of some humidity sensors reported in the literature.

Sensing Material	Range RH (%)	Humidity Response (%)	Hysteresis (%)	Response/Recovery Times (s)	Operating Frequency	Reference
BiFeO_3_	17–94	5.8 × 10^3^	0.52	165/40	100 Hz	Present work
20% ZnO-BiFeO_3_	17–94	2.8 × 10^6^	2.14	98/64	100 Hz	Present work
TiO_2_	11–95	3.5 × 10^3^	4.66	3/50	100 Hz	[13]
BiFeO_3_	16–92	4.6 × 10^3^	3.0	50/150	1 kHz	[31]
BiFeO_3_/Carbon fibers	16–92	1.2 × 10^4^	3.5	130/75	1 kHz	[31]
ZnO Nanorods	33–95	61.23			200 kHz	[37]
ZnO-SnO_2_	32–92	95	2.9	17/65	100 Hz	[43]
Graphite-oxyde	11–97	4.4 × 10^6^		3/5	10 Hz	[66]
ZnO-polyethylenimine	15–95	43.9 × 10^6^	8	5/3	100 Hz	[67]

**Table 2:** Time-dependent evolution of RSD for various RH values ranging from 17% to 94% for a BFO-based sensor.

Time (Days)	RH = 17%	RH = 25%	RH = 35%	RH = 42%	RH = 56%	RH = 68%	RH = 75%	RH = 87%	RH = 94%
0	0	0	0	0	0	0	0	0	0
10	0	0	0	1.19	0	−1.68	0.18	0	0.16
20	−5.90	−2.50	1.70	3.09	−0.80	−2.52	−2.39	−2.40	0.35
30	0	2.50	2.97	4.52	0.33	−5.46	−1.65	0.37	0.69
40	−4.10	4.00	3.22	5.95	1.33	−4.41	0.18	0.48	0.99
50	−3.59	4.50	4.21	7.14	2.44	−1.47	0.99	0.61	1.27
60	−2.56	4.25	4.71	6.42	2.22	−2.31	−1.10	0.75	1.50
70	−2.56	4.00	4.96	3.57	2.20	−1.26	2.28	−1.80	1.81
80	−5.90	3.75	5.45	7.14	1.98	0.84	3.13	−1.80	1.93
90	−1.79	3.75	6.45	5.95	1.75	2.94	2.20	−1.66	2.17
100	−1.79	4.50	6.69	4.76	1.31	7.14	4.97	−0.18	2.50
120	−1.28	4.75	6.94	4.52	1.33	8.19	7.73	1.28	2.73
140	−0.51	5.00	7.69	4.04	1.11	10.29	4.97	2.63	2.98
160	0	5.00	8.18	3.73	0.89	9.24	7.55	3.51	3.20
180	1.28	5.00	8.68	3.50	0.44	10.50	9.76	6.79	3.57
200	2.82	5.25	8.93	3.09	0.22	11.34	12.33	7.73	3.20

**Table 3:** Time-dependent evolution of RSD for various RH values ranging from 17% to 94% for a 20% ZnO-BFO-based sensor.

Time (Days)	RH = 17%	RH = 25%	RH = 35%	RH = 42%	RH = 56%	RH = 68%	RH = 75%	RH = 87%	RH = 94%
0	0	0	0	0	0	0	0	0	0
10	0.48	−0.41	−0.47	−0.43	0.39	−0.26	0.35	3.10	−5.92
20	−0.16	0.30	−0.38	2.32	−3.94	0.11	−0.19	−1.12	−6.92
30	0.80	0	−0.19	5.33	3.69	0.28	−0.53	−1.18	−7.30
40	0.32	−0.81	0.09	10.00	−4.11	0	−2.13	−5.29	−6.40
50	0.64	−0.20	2.24	7.39	−1.16	7.05	−7.46	−8.97	−8.37
60	1.13	0.10	2.29	10.4	6.88	6.90	−5.33	−6.95	−5.70
70	−1.61	0.60	2.62	10.384	2.17	7.46	−7.25	−8.32	−8.49
80	1.51	0.71	2.72	9.77	10.74	7.49	−8.05	−4.98	−8.50
90	3.22	1.12	8.40	−1.14	−1.72	6.86	−7.76	−1.35	−5.90
100	4.99	0.81	6.97	1.81	−1.68	4.77	0.77	−4.71	−3.50
120	7.41	−1.93	5.06	5.43	−5.67	4.61	−4.45	−0.99	−3.55
140	8.86	−1.72	4.58	7.44	−1.97	4.53	0.90	−4.34	−3.54
160	6.92	−2.34	6.97	10.67	2.05	4.75	3.28	3.12	−3.55
180	10.30	−1.52	5.53	13.25	6.97	4.44	0.61	2.37	−5.68
200	12.72	−1.12	4.10	13.80	10.73	2.03	−3.23	7.14	−7.05

## Data Availability

The data are available on request from the authors.
